# Sydenham Society

**Published:** 1847-07

**Authors:** 


*Sydenham Society.*—The Society held its fourth anniversary meeting on Saturday, May 1st, Dr. Latham in the chair. The report was read by the Secretary, Dr. Risdon Bennet, and represented the Society, as in a flourishing condition. A complete edition of Hippocrates was stated to be in progress, as also a work on medical physiology, and another on medical ethics. A work on ancient medical Bibliography was also in progress. The Treasurer read his report from which it appeared that there was a balance in hand of £700.—*Prov. Med. & Surg. Jour.*

